# Weight Change after Striatal/Capsule Deep Brain Stimulation Relates to Connectivity to the Bed Nucleus of the Stria Terminalis and Hypothalamus

**DOI:** 10.3390/brainsci9100264

**Published:** 2019-10-03

**Authors:** Juan Carlos Baldermann, Lisa Hahn, Till A. Dembek, Sina Kohl, Jens Kuhn, Veerle Visser-Vandewalle, Andreas Horn, Daniel Huys

**Affiliations:** 1 Department of Psychiatry and Psychotherapy, University Hospital of Cologne, Medical faculty, 50937 Cologne, Germany; sina.kohl@uk-koeln.de (S.K.); jens.kuhn@uk-koeln.de (J.K.); daniel.huys@uk.koel.de (D.H.); 2 Institute of Neuroscience and Medicine, Brain & Behaviour (INM-7), Research Centre Jülich, 52428 Jülich, Germany; l.hahn@fz-juelich.de; 3 Institute of Systems Neuroscience, Medical Faculty, Heinrich Heine University Düsseldorf, 40225 Düsseldorf, Germany; 4 Department of Neurology, University Hospital of Cologne, Medical faculty, 50937 Cologne, Germany; till.dembek@uk-koeln.de; 5 Johanniter Hospital Oberhausen, Department of Psychiatry, Psychotherapy and Psychosomatics, 46145 Oberhausen, Germany; 6 Department of Stereotactic and Functional Neurosurgery, University of Cologne, 50937 Cologne, Germany; Veerle.visser-Vandewalle@uk-koeln.de; 7 Department of Neurology, Charité-University Medicine (CVK), 10117 Berlin, Germany; andreas.horn@charite.de

**Keywords:** deep brain stimulation, DBS, weight, food intake, bed nucleus of the stria terminalis, obesity, obsessive-compulsive disorder, addiction

## Abstract

Weight changes are insufficiently understood adverse events of deep brain stimulation. In this context, exploring neural networks of weight control may inform novel treatment strategies for weight-related disorders. In this study, we investigated weight changes after deep brain stimulation of the ventral striatum/ventral capsule and to what extent changes are associated with connectivity to feeding-related networks. We retrospectively analyzed 25 patients undergoing deep brain stimulation for obsessive-compulsive disorder or substance dependency. Weight changes were assessed preoperatively and six to twelve months after surgery and then matched with individual stimulation sites and stimulation-dependent functional connectivity to a priori defined regions of interest that are involved in food intake. We observed a significant weight gain after six to twelve months of continuous stimulation. Weight increases were associated with medial/apical localization of stimulation sites and with connectivity to hypothalamic areas and the bed nucleus. Thus, deep brain stimulation of the ventral striatum/ventral capsule influences weight depending on localization and connectivity of stimulation sites. Bearing in mind the significance of weight-related disorders, we advocate further prospective studies investigating the neuroanatomical and neuropsychological underpinnings of food intake and their neuromodulatory therapeutic potential.

## 1. Introduction

Deep brain stimulation (DBS) is an established treatment for movement disorders such as Parkinson’s disease [1]. More recently, DBS targeting the ventral striatum (VS) with the nucleus accumbens (NA) and the ventral part of the anterior limb of the internal capsule (ALIC), also referred to as ventral capsule (VC), has been employed for the treatment of patients with obsessive-compulsive disorder (OCD) and addiction [2,3]. Interestingly, both weight gain and loss have been reported as potential adverse events [2]. DBS of the VS/VC has indeed been considered as a potential target for treating anorexia nervosa [4,5]. In contrast, the NA has also been considered a potential DBS target for treating obesity due to case reports partly reporting drastic weight loss [6,7,8]. Notably, there is scarce systematic data on the effects of VS/VC DBS on weight. In a retrospective study, Linssen and colleagues [9] did not observe weight change after DBS of the ALIC. In this study, data from different time points of observation for each patient were pooled, whereby the follow-up period ranged from 10 months to 8.7 years, making it difficult to draw clear conclusions from the study. In summary, the literature is highly inconsistent regarding the relationship between VS/VC stimulation and weight change. 

Apart from direct stimulation of certain nuclei or fiber bundles, changes in weight may result from indirect modulation of broader networks [10]. Recently, Luo et al. [11] provided breakthrough insights into the neural regulation of feeding and its neuromodulatory potential by showing that selective excitatory optogenetic stimulation of GABAergic somatostatin neurons of the tuberal nucleus within the lateral hypothalamic area (LHA) in mice led to enhanced food intake via connections to the bed nucleus of the stria terminalis (BNST) and paraventricular nucleus (PVN). Hence, DBS of the VS/VC may interfere with eating and hunger via functional connections to said network.

Based on the conflicting literature and the recently gained insights into neural networks associated with food intake, we investigated changes in weight during six to twelve months of VS/VC DBS. Secondly, we examined to what extent weight changes are associated with individual neuroanatomical positioning of DBS electrodes anvatd corresponding electric fields as well as functional connectivity to the abovementioned network.

## 2. Materials and Methods

### 2.1. Subjects

Twenty-five patients (mean age = 45.4 ± 12.3, 14 women) with treatment-refractory OCD (*n* = 20) or addiction to alcohol or opioids (*n* = 5), who underwent deep brain stimulation in the VS/VC between 2010 and 2016 at the University Hospital Cologne, were retrospectively included in the study. Inclusion criteria were: (1) Preoperative magnetic resonance imaging (MRI) and postoperative MRI or computer tomography (CT) scan was available and (2) the weight of the patient was measured preoperatively and 6 to 12 months after surgery. All patients consented to the procedure according to the Declaration of Helsinki. The study was approved by the Ethics Committee of the Medical Faculty of the University of Cologne.

### 2.2. Surgical Procedure

Under local or general anesthesia, quadripolar electrodes (Model 3387 DBS Lead; Medtronic; Minneapolis, MN, USA) were stereotactically implanted bilaterally. The two distal contacts (0,1 on the left and 8,9 on the right electrode, respectively) were placed in the NA bilaterally. The more proximal contacts (2,3 and 10,11) were located in the ventral part of the VC. Stereotactic X-ray procedures in the operating room and postoperative CT were used to confirm the correct position of the electrodes postoperatively. For further description of surgical procedure and patient inclusion criteria for OCD patients, see [12].

### 2.3. Reconstruction of Volume of Tissue Activated and Connectivity Analysis

DBS electrodes and volumes of tissue activated (VTA) were reconstructed in Lead-DBS (V 2.0, https://www.lead-dbs.org) [13]. To summarize, postoperative MRI or CT scans were linearly coregistered to preoperative MRI. Coregistered images were then manually controlled for each subject and refined if needed using Advanced Normalization Tools (http://stnava.github.io/ANTs/). Subsequently, images were normalized into the International Consortium of Brain Mapping (ICBM) 2009b non-linear asymmetric space using the symmetric normalization approach implemented in Advanced Normalization Tools. A subcortical brain shift correction was applied using the Lead-DBS software if needed to attain a more precise subcortical alignment. DBS electrodes were localized within the Montreal Neurological Institute (MNI) space by identifying the artifact in the postoperative image using the Lead-DBS software. The VTA estimation protocol followed the one used by Horn and colleagues [14]. Left-hemispherical VTAs were then flipped non-linearly to the right side to create averaged maps by pooling VTAs and averaging corresponding weight changes for each voxel which was modulated by at least 10% of the patients. Additionally, individual bilateral VTAs were used as seeds to calculate stimulation-dependent functional connectivity based on publicly available normative resting state functional connectivity [15] to a priori defined regions of interest (ROI) as described by Horn et al. [14]. To calculate normative stimulation-dependent connectivity, publicly available data from the Brain Genomics Superstruct Project (https://dataverse.harvard.edu/dataverse/GSP) were used, including resting state functional connectivity data obtained on 1000 healthy subjects using a 3T Siemens (Erlangen, Germany) MRI system. Preprocessing included global signal regression and spatial smoothing at 6 mm full-width at half maximum. Consequently, functional connectivity estimates were calculated within Lead-DBS between bilateral VTAs and ROIs. ROIs were derived from the aforementioned feeding network proposed by Luo et al. [11] encompassing the BNST, LHA, and PVN. A paired t-test for dependent samples was calculated for weight changes and Pearson’s coefficient for correlation analysis of functional connectivity estimates and weight changes. We assessed false discovery rate (FDR) adjusted p-values to correct for multiple testing in the correlation analysis. Statistical analysis was performed in SPSS (IBM Corp. Version 25.0. Armonk, NY, USA).

## 3. Results

**Figure 1:**
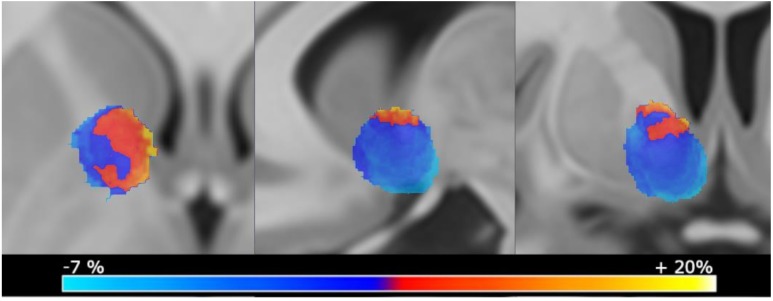
Averaged weight change maps displaying mean weight changes per voxel. Only voxels that were stimulated by at least 10% of the patients were selected to control for outliers. Volumes of activated tissue (VTA) that were located more medially and apically were associated with more weight gain after intervention.

We found a significant mean weight increase of 4.6 kg ± 9.6 (t(24) = −2.4, *p* = 0.024) from baseline (86.4 kg ± 20.4) to six to twelve months after surgery (91.0 kg ± 22.1) (Figure 1). Of note, two patients experienced excessive weight gain above 25% from baseline (27.4% and 39.5%). An additional review of weight changes at last available follow-up (see Table 1) showed that nine out of 14 patients in which such data was available showed a lasting increase in weight compared to baseline. This implied that weight gains were not only present temporarily in these patients. Within the OCD group, there was no correlation between weight change and percentage symptom reduction (*r* = 0.002; *p* = 0.992) (Figure S1). We did not perform this analysis for the addiction subgroup since only five patients were included and outcome was not assessed uniformly.

Imaging analysis revealed that more medially and apically located VTAs in proximity to the BNST were associated with the most extensive weight gain (Figure 1).

**Figure 2:**
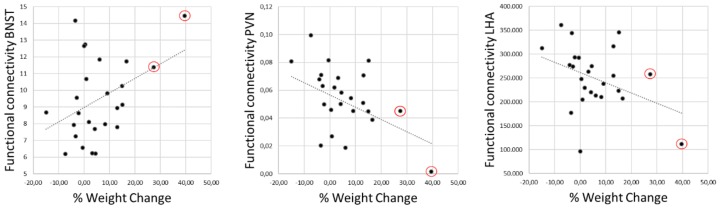
Pearson’s correlation analysis of weight changes with functional connectivity estimates between individual stimulation volumes and bed nucleus of the stria terminalis (BNST), paraventricular nucleus (PVN), and lateral hypothalamic area (LHA). All regions revealed significant correlations with weight changes (BNST: *r* = 0.420, *p* = 0.018; PVN: *r* = –0.405, *p* = 0.016; LHA: *r* = −0.384, *p* = 0.028) that remained significant after correction for multiple comparisons. Of note, two patients experienced an excessive weight gain of over 25% (marked in red circles).

Furthermore, we observed significant correlations between weight change and functional connectivity estimates for BNST (*r* = 0.420; *p* = 0.018; *p*_FDR_ = 0.027) and conversely, significant anticorrelation for LHA (*r* = −0.384; *p* = 0.028; *p*_FDR_ = 0.028) and PVN (*r* = −0.405; *p* = 0.016; *p*_FDR_ = 0.027) (Figure 2).

## 4. Discussion

In this retrospective study, we aimed to shed some light on weight changes after DBS of the VC/VS for OCD and addiction. Our analysis showed that VS/VC DBS was indeed associated with an increase in weight with a significant mean increase of 4.6 (± 9.5) kg after six to twelve months of ongoing DBS. Strikingly, two patients showed an excessive weight gain of over 25%. An exploratory examination of weight development to the last available follow-up indicated that this weight gain was not a temporary phenomenon. Hence, weight change is a relevant adverse event of DBS of the ventral striatum, that should be carefully monitored by clinicians. Furthermore, analysis of VTA locations and functional connectivity indicate that a network comprising the BNST and hypothalamic regions is involved in DBS‑related weight changes. Thus, congruent to animal research [11], electrical modulation of a network comprising hypothalamic areas and the BNST can potentially induce changes in weight in humans.

Our results raise the question if weight changes come along with symptom reduction after DBS. However, we did not observe a statistically significant correlation between weight changes and changes in obsessive-compulsive symptoms. This indicates that weight gain forms an independent side effect of the stimulation. In fact, our VTA analysis showed that weight gain was associated mostly with medially located VTAs encompassing the BNST and the dorsomedial NA. In a recent analysis that included a similar VTA analysis, OCD symptom reduction was mainly associated with a distinct fiber tract within the ALIC that passed slightly above the observed hot spot for weight gain [16]. This portends that weight gain is mediated through a distinct network. Further disentanglement of the neuroanatomical equivalents of adverse events and actual DBS-related symptom reduction might lead to optimized targeting in future, potentially reducing undesirable effects such as weight gain.

The mechanisms that underlie DBS-associated weight increase remain ambiguous. One possibility on how DBS of the VC/VS may influence weight is through altering homeostatic control in the hypothalamus. The hypothalamus constitutes a key junction between the endocrine and nervous systems by integrating feeding-related sensory signals providing close connections with brainstem regions involved in autonomic monitoring [17]. Moreover, the LHA region is implicated in hunger and satiety [18]. The PVN is involved in the regulation of leptin, a hormone that regulates energy balance and hunger [19]. Similarly, the BNST is connected to the LHA. Inhibitory inputs from the BNST control food intake by innervating or suppressing the activity of glutamatergic neurons in the LHA [20]. Consequently, the BNST possibly influences the LHA to control food. Thus, by modulating this functionally connected area, DBS may directly change hunger and satiety.

Another possibility on how DBS of the VC/VS may influence weight is through impaired impulse control i.e., increased impulsivity or reward sensitivity. Increased impulsivity or increased reward sensitivity towards food intake is a key factor in obesity [21]. In this context, increased impulsivity and disinhibition have been reported repeatedly as adverse effects of DBS for OCD [4]. It is conceivable that by influencing impulse control through DBS of the VC/VS, patients may be more prone to follow eating impulses. Another possible mechanism of action for how DBS of the VS/VC may change weight is through altered reward processing. The VS is significantly involved in reward and motivation. With regard to food intake, the VS mediates the hedonic and motivational aspects of food reward [22]. The finding that VTAs located in proximity to the dorsomedial striatum relate to the most pronounced weight changes is particularly interesting, since this region—at least in animal research—is specifically important for hedonic effects associated with food intake (e.g., the desire for sugar) [23]. Specifically, animal studies showed that bilateral modulation of the NA is functionally dissociated between the lateral and medial shell. Whereas stimulation of the lateral shell decreased the motivation to work for palatable food, stimulation of the medial shell increased the food intake [24]. Additionally, the LHA interacts with the NA via orexinergic connections, thereby regulating hedonic and motivational incentive salience [22]. Thus, both NA and LHA are implicated in motivational aspects of food intake. A potential mechanism of DBS-related weight gain may thus be altered motivation to eat mediated by the NA, BNST, and LHA.

Functional MRI (fMRI) studies in humans have shown that the VS/NA, BNST, and LHA form a functionally connected network that is altered in subjects with obesity [25,26]. Hence, the VS/NA, BNST, and LHA form a functionally connected network that regulates rewarding aspects of food intake, and VC/VS DBS putatively modulates this network. Our results suggest that interfering with formerly balanced functional connectivity between VS/NA, BNST, and hypothalamic nuclei results in weight gain. Whether VS/VC DBS influences food intake via changes in impulsivity or reward processing, or if homeostatic changes resulting from decoupled striatal input underlie this finding constitutes an important research question for further studies.

It is important to take into consideration the overt limitations of the retrospective character of the study. Several factors potentially influenced weight, which we were not able to track in our sample. First, temporary weight changes may be influenced by lifestyle changes such as exercise, diet, or eating habits following DBS. We are not aware that patients in our sample consciously changed these factors, but cannot rule them out. Second, an important potentially confounding factor is changes in medication. Augmentation therapy with antipsychotics in particular poses a higher prevalence of metabolic syndrome in patients with OCD associated with the duration of the exposure to antipsychotics [27]), while L-polamidone used in the treatment of opioid dependence can lead to reduced appetite and weight loss. Our patients tend to keep medication stable and if anything, patients tend to reduce potentially weight-enhancing psychotropic drugs after DBS which might actually mask some of the DBS‑related weight gain. Third, successful treatment of baseline symptoms might influence weight (e.g., reduced calorie-intake by abstinence from alcohol). We did not find such an association with OCD symptom reduction but did not test this association for dependency, as the outcomes were not comparable and the analysis would have included only five patients. Despite the limitations, we argue that our results are of particular importance, both to explain potential side effects of VS/VC DBS and to suggest a potential approach to modulate food intake. First, we could show DBS of the VS/VC is indeed associated with significant weight gain that in individual cases can result in extensive weight gain. Second, we could show that weight changes depend on the localization of electrodes and corresponding VTAs. Third, weight gain can be linked to a broader functional network that is implicated in control of food intake. Bearing in mind the significance of weight-related disorders, we advocate further prospective studies investigating the biological and neuropsychological underpinnings of weight changes and their neuromodulatory therapeutic potential.

## Figures and Tables

**Table 1:** Demographic data and weight changes.

SUBJECT	AGE	SEX	DIAGNOSIS	BMI PRE-DBS	WEIGHT PRE-DBS (KG)	WEIGHT POST-DBS (KG)	WEIGHT AT LAST FOLLOW-UP (MONTHS AFTER DBS)
1	52	f	OCD	25	67.00	57.00	55.00 (20)
2	31	m	OCD	32	113.20	104.80	114.30 (41)
3	40	f	OCD	18	49.00	47.00	48.10 (35)
4	56	f	OCD	25	74.60	72.00	72.00 (16)
5	31	f	OCD	34	121.00	117.00	NA
6	27	m	ADD	31	92.60	90.00	NA
7	39	f	OCD	29	87.90	86.00	NA
8	57	m	ADD	41	126.70	126.00	126.00 (16)
9	34	m	ADD	27	88.40	88.40	NA
10	56	m	OCD	31	97.50	98.00	NA
11	46	m	OCD	24	81.90	82.60	82.30 (19)
12	39	f	OCD	21	54.00	55.00	63.00 (23)
13	37	f	OCD	37	108.10	111.60	NA
14	66	m	OCD	27	88.60	92.40	NA
15	34	f	OCD	31	78.10	81.60	NA
16	28	m	OCD	28	88.50	93.90	100.00 (19)
17	60	f	OCD	32	97.00	105.00	NA
18	38	f	OCD	26	77.00	84.00	NA
19	67	f	OCD	28	80.00	90.40	75.50 (42)
20	52	m	ADD	20	69.00	78.00	78.00 (19)
21	49	m	OCD	27	85.00	97.70	92.00 (20)
22	62	m	OCD	37	120.30	138.50	134.10 (35)
23	39	f	ADD	21	56.60	66.00	NA
24	37	f	OCD	30	73.00	93.00	89 (20)
25	57	f	OCD	32	86.00	120.00	122.1 (24)

Weight was tracked before (pre-DBS) and after 6–12 months (post-DBS) of continuous deep brain stimulation. If available, we added weight data at last follow-up with duration from surgery indicated in brackets. ADD = addiction; OCD = obsessive-compulsive disorder; f = female; m = male; NA = date not available.

## Supplementary Materials

The following are available online at https://www.mdpi.com/2076-3425/9/10/264/s1, Figure S1: Scatter plot of weight changes after deep brain stimulation and changes in symptoms of obsessive-compulsive disorders assessed using the Yale-Brown Obsessive Compulsive Scale (Y-BOCS).
